# Recent studies in microbial degradation of petroleum hydrocarbons in hypersaline environments

**DOI:** 10.3389/fmicb.2014.00173

**Published:** 2014-04-23

**Authors:** Babu Z. Fathepure

**Affiliations:** Department of Microbiology and Molecular Genetics, Oklahoma State UniversityStillwater, OK, USA

**Keywords:** hypersaline environments, biodegradation, oxygenated and non-oxygenated hydrocarbons, halophilic and halotolerant bacteria and archaea, molecular mechanism of degradation

## Abstract

Many hypersaline environments are often contaminated with petroleum compounds. Among these, oil and natural gas production sites all over the world and hundreds of kilometers of coastlines in the more arid regions of Gulf countries are of major concern due to the extent and magnitude of contamination. Because conventional microbiological processes do not function well at elevated salinities, bioremediation of hypersaline environments can only be accomplished using high salt-tolerant microorganisms capable of degrading petroleum compounds. In the last two decades, there have been many reports on the biodegradation of hydrocarbons in moderate to high salinity environments. Numerous microorganisms belonging to the domain Bacteria and Archaea have been isolated and their phylogeny and metabolic capacity to degrade a variety of aliphatic and aromatic hydrocarbons in varying salinities have been demonstrated. This article focuses on our growing understanding of bacteria and archaea responsible for the degradation of hydrocarbons under aerobic conditions in moderate to high salinity conditions. Even though organisms belonging to various genera have been shown to degrade hydrocarbons, members of the genera *Halomonas Alcanivorax*, *Marinobacter*, *Haloferax*, *Haloarcula*, and *Halobacterium* dominate the published literature. Despite rapid advances in understanding microbial taxa that degrade hydrocarbons under aerobic conditions, not much is known about organisms that carry out similar processes in anaerobic conditions. Also, information on molecular mechanisms and pathways of hydrocarbon degradation in high salinity is scarce and only recently there have been a few reports describing genes, enzymes and breakdown steps for some hydrocarbons. These limited studies have clearly revealed that degradation of oxygenated and non-oxygenated hydrocarbons by halophilic and halotolerant microorganisms occur by pathways similar to those found in non-halophiles.

## Background

Many hypersaline environments including natural saline lakes, salt flats, saline industrial effluents, oil fields, and salt marshes are often contaminated with high levels of petroleum hydrocarbons. These systems have considerable economic, ecological and scientific value. Among the contaminated hypersaline environments, oilfields pose a special problem due to their sheer numbers all over the world and due their high salinity caused by salty brackish water (produced water) generated during oil and natural gas extraction. Produced waters are by far the largest volume byproduct or waste associated with oil and gas production. For every barrel of oil produced, roughly 10 barrels of produced waters are generated. In the United States about 20–30 billion barrels of produced waters are generated each year and the worldwide estimate is about 70 billion barrels per year (Veil et al., 2004). The primary constituents in produced water that limit its disposal or reuse are high levels of salt (1000–250,000 mg/L), oil and grease, various toxic chemicals, heavy metals, and naturally occurring radioactive materials (Veil et al., 2004; Cuadros-Orellana et al., 2006; Bonfá et al., 2011).

Remediation of produced water is costly to oil and gas producers and inappropriate management can lead to environmental problems. Presently, >95% of all produced waters are re-injected, however prior to 1965–1970 most of the produced water waste was released to the surface. Even now many small- to moderate-sized operators continue to release substantial quantities of produced waters to the surface and shallow subsurface because of leaky tanks and flow-lines and due to accidents and vandalism. Sabkhas or coastal salt marshes are ubiquitous features in arid and semi-arid regions of the world (Arabian Peninsula, Central Asia, and Australia). These habitats are characterized by high salinity and extensive crude oil contamination (Fowler et al., 1993; Al-Mueini et al., 2007; Al-Mailem et al., 2013). Understanding the fate of petroleum compounds in such environmentally and economically sensitive habitats is important.

Bioremediation technology utilizes microorganisms to degrade toxic pollutants to harmless products such as CO_2_, H_2_O, and other inorganic compounds and these processes are environmentally safe and cost efficient (Philip et al., 2005). It has been reported that roughly 25% of all petroleum-contaminated land is being bioremediated using natural attenuation processes thus underscoring the importance of microorganisms in remediation strategies (Holden et al., 2002). However, application of microbial technologies for treating contaminated high salinity or fluctuating salinity environment is limited due to the detrimental effects of salt on microbial life including disruption cell membrane, denaturation of enzymes, low solubility of oxygen, low solubility of hydrocarbons, and desiccation (Pernetti and Di Palma, 2005). Therefore, bioremediation of saline environments without costly dilution of salt-laden soil and water requires halophilic or halotolerant organisms that tolerate high salt concentrations. Halophiles are classified into three groups according to their optimal salt concentration for growth: slightly halophilic (1–3% w/v), moderately halophilic (3–15% w/v), and extremely halophilic (15–32% w/v) (Kushner, 1978; Ventosa and Nieto, 1995; Oren, 2013).

## Degradation of hydrocarbons in hypersaline environments

Petroleum is a complex mixture of different hydrocarbons including aliphatic (linear or branched), cycloalkanes, mono- and polyaromatics, asphaltenes and resins and majority of these compounds are stable, toxic, and carcinogenic (Philip et al., 2005; Yemashova et al., 2007). Hydrocarbons differ in their susceptibility to microbial attack and generally degrade in the following order of decreasing susceptibility: n-alkanes > branched alkanes > low molecular weight aromatics >cyclic alkanes, > polyaromatic hydrocarbons (Leahy and Colwell, 1990). Although many of these compounds can be relatively easily degraded under soil and freshwater environments (Van Hamme et al., 2003; Cao et al., 2009) and low salinity marine habitats (Harayama et al., 1999; Head and Swannell, 1999; Head et al., 2006; McGenity et al., 2012), little is known about their fate in moderate to high salinity conditions (3–30% salt). In 1992 Oren (Oren et al., 1992) provided an overview of the degradation of aromatic and aliphatic hydrocarbons in saline habitats and our understanding of metabolic capabilities of halophilic and halotolerant organisms has substantially advanced since this publication (Patzelt, 2005). For example, recent excellent reviews by Le Borgne et al. (2008), Martins and Peixoto (2012), McGenity (2010), and Patzelt (2005) attest to our improved understanding of the hydrocarbon biodegradation by halophilic and halotolerant microorganisms. Nonetheless, our knowledge on biochemistry, genetics, and pathways of hydrocarbon degradation in halophiles and halotolerants is sparse. Such information is crucial for designing novel and more efficient technologies for the remediation of contaminated high salinity environments and for understanding the carbon cycle in such extreme habitats. The goal of this review is to provide an overview of our current knowledge of the biodegradation of non-oxygenated and oxygenated hydrocarbons by bacteria and archaea in wide ranging salinities (6–30% NaCl) and to highlight recent discoveries in molecular mechanisms of degradation by halophilic and halotolerant organisms.

### Crude oil

Crude oil is a mixture of hydrocarbons composed of mainly oxygenated and non-oxygenated hydrocarbons (Yemashova et al., 2007). To date many studies have reported the ability of microorganisms to utilize crude oil components as the growth substrates in moderate to high salinity environments (Table 1). Diaz et al. (2000) have enriched microbial consortia, MPD-7 and MPD-M from Cormorant oil fields in North Sea and sediments associated with mangrove roots, respectively. These cultures degraded aliphatic and aromatic hydrocarbons in crude oil at salinity ranging from 3.5 to 10% NaCl. Total oil degradation by MPD-7 ranged from 20 to 38%, while MPD-M degraded much higher amount of crude oil ranging between 45 and 48%. In a subsequent study, Diaz et al. (2002) have immobilized the MPD-M culture on polypropylene fibers and showed that the culture was able to degrade crude oil at much higher salinity up to 18% NaCl. Riis et al. (2003) were able to show the degradation of diesel fuel in the presence of salt up to 17.5% by microbial communities extracted from Argentinean saline soils. In addition, these investigators isolated several halotolerant bacteria of the genera *Cellulomonas*, *Bacillus*, *Dietzia*, and *Halomonas* with the ability to degrade crude oil as the carbon source. Similarly, many other investigators have isolated pure cultures including *Halomonas shengliensis* (Wang et al., 2007), *Halomonas* sp. strain C2SS100 (Mnif et al., 2009), *Marinobacter aquaeolei* (Huu et al., 1999), *Streptomyces albiaxialis* (Kuznetsov et al., 1992), *Rhodococcus erythropolis*, and *Dietzia maris* (Zvyagintseva et al., 2001) from oilfields, production water, and other saline environments that degrade crude oil as the source of carbon in the presence of 0–30% salt. Borzenkov et al. (2006) reported the isolation of several strains of hydrocarbon-oxidizing bacteria representing the genera *Rhodococcus, Gordonia, Dietzia*, and *Pseudomonas* from oil and stratal waters of Tatarstan, western Siberia, and Vietnam oilfields. All these strains oxidized *n*-alkane fraction of crude oil in a medium containing 15% NaCl. A *Bacillus* sp. strain DHT, isolated from oil contaminated soil, grew and produced biosurfactant when cultured in the presence of variety of hydrocarbons including crude oil, diesel oil, hexadecane, naphthalene, pyrene, dibenzothiophene, salicylate, catechol, and phenanthrene as the sole sources of carbon in the presence of 0–10% salinity and at 30–45°C. However, no growth occurred on toluene, phenol, 2-hydroxyquinoline and carbazole (Kumar et al., 2007). Similarly, Mnif et al. (2011) have reported the isolation of several strains of thermophilic and mesophilic hydrocarbon degrading as well as biosurfactant producing organisms from Tunisian oil fields. Among these, *Pseudomonas* sp. strain C450R and *Halomonas* sp. strain C2SS100 could degrade 93–96% of the aliphatic fraction of crude oil (C_13_–C_29_), while producing biosurfactants in the presence of 5–10% NaCl. Such organisms could play important role in the degradation of poorly soluble high molecular weight hydrocarbons in crude oil. Chamkha et al. (2008) have isolated a strain C5 closely related to *Geobacillus pallidus* from a tyrosol degrading enrichment developed from production water from a high-temperature oil field in Tunisia. The organism degraded crude oil and diesel as the source of carbon in the presence of 0–12% NaCl. Wang et al. (2010) have isolated a moderate halophilic actinomycete, *Amycolicicoccus subflavus* DQS3-9A1^*T*^ from oily sludge at Daqing Oilfield, China with the ability to degrade crude oil in the presence of 1–12% NaCl. Later, Nie et al. (2013) studied the genetic capability of the DQS3-9A1^*T*^ to metabolize a range of short-chain and long-chain *n*-alkanes such as propane and C_10_–C_36_ alkanes, respectively, as the sole carbon sources in the presence of 1–12% NaCl. Recently, Al-Mailem et al. (2013) have isolated *Marinobacter sedimentalis* and *Marinobacter falvimaris* from soil and pond water collected from hypersaline sabkhas (18–20% salinity) in Kuwait. Isolation of these organisms was accomplished using agar plates provided with crude oil vapor as the sole source of carbon and 6% NaCl. These studies also showed that both organisms were capable of fixing atmospheric nitrogen and such potential is beneficial for effective bioremediation of petroleum compounds at high salinity without the need of providing fertilizer.

**Table 1:** **Biodegradation of crude oil under moderate to high salinity environment**.

**Hydrocarbon**	**Degrader**	**Salinity (%)**	**References**
Crude oil	*Streptomyces albiaxialis*	3–30	Kuznetsov et al., 1992
	Enrichment culture, brines of the Kalamkass oil fields, Kazakhastan	10–25	Zvyagintseva et al., 1995
	*Marinobacter aquaeolei*	0–20	Huu et al., 1999
	Bacterial consortia MPD-7	3.5–10	Diaz et al., 2000
	*Rhodococcus erythropolis Dietzia maris*	0–10	Zvyagintseva et al., 2001
	MPD-M culture immobilized on polypropylene fibers	0–18	Diaz et al., 2002
	*Fusarium lateritium Drechslera* sp.	5–10	Obuekwe et al., 2005
	Microbial community, Argentinean soil	>17	Riis et al., 2003
	*Cellulomonas* sp. *Bacillus* sp. *Dietzia* sp. *Halomonas* sp.	>17	Riis et al., 2003
	*Rhodococcus* sp. *Gordonia* sp. *Dietzia* sp. *Pseudomonas* sp.	15	Borzenkov et al., 2006
	*Actinopolyspora* sp. DPD1	20	Al-Mueini et al., 2007
	*Bacillus* sp. strain DHT	0–10	Kumar et al., 2007
	*Halomonas shengliensis*	0–15	Wang et al., 2007
	Strain C5	12	Chamkha et al., 2008
	*Halomonas* sp. C2SS100 *Pseudomonas* sp. C450R	0–10	Mnif et al., 2009, 2011
	*Haloferax* sp. *Halobacterium* sp. *Halococcus* sp.	>26	Al-Mailem et al., 2010
	*Amycolicicoccus subflavus* DQS3-9A1	1–12	Wang et al., 2010
	*Marinobacter sedimentalis*, *Marinobacter falvimaris*	6	Al-Mailem et al., 2013

Studies also have reported archaeal ability to degrade crude oil in hypersaline environments. Zvyagintseva et al. (1995) have reported that a significant amount of isoprenoid and *n*-alkane fractions of crude oil was degraded in the presence of 10–25% of salt by an enrichment developed from the brines of the Kalamkass oil fields in Kazakhstan. Al-Mailem et al. (2010) have isolated extremely halophilic archaeal strains of *Haloferax*, *Halobacterium*, and *Halococcus* from a hypersaline coastal area of the Arabian Gulf in a mineral salt medium with crude oil vapor as the source of carbon in the presence of >26% NaCl and at 40–45°C. These organisms also metabolized various aliphatic and aromatic hydrocarbons as the sole sources of carbon and energy at high salinity. Undoubtedly such properties are important for the bioremediation of crude oil-impacted high salinity arid sites. In a subsequent study by some of the same authors, the impact of adding organic fertilizer (casamino acid) and illumination (light/dark) on the bioremediation of crude oil was assessed using hypersaline soil (>22% salinity) and pond water (>16% salinity) collected from a supertidal sabkha at Al-Khiran, Kuwait. Results showed a significantly increased biodegradation of crude oil in the presence of casamino acid and when incubated under continuous illumination (Al-Mailem et al., 2012). The data suggested that the observed increased degradation was mainly due to archaeal members, with little or no contribution from bacteria. The authors theorize that hypersaline environments suffer from the lack of oxygen due low solubility and archaea in such environments would use the red pigment-mediated ATP synthesis perhaps analogous to bacteriorhodopsin-like system to meet the shortage of ATP produced *via* oxidative phosphorylation caused by low oxygen tension. This strategy would allow archaea to utilize the available limited oxygen to initiate degradation of hydrocarbons in high salinity conditions. In addition, the authors contend that casamino acid could have been used as the source of amino acids resulting in better growth and degradation. In conclusion, the enhanced hydrocarbon degradation in the presence of light is an interesting observation and warrants further investigation into why archaea dominate hypersaline environments. In return, such knowledge could be helpful to develop strategies to enhance hydrocarbon degradation in high salinity environments.

Only few studies are available on the fungal ability to degrade hydrocarbons in high salinity environments. Obuekwe et al. (2005) are the first to report the isolation *Fusarium lateritium*, *Drechslera* sp, and *Papulaspora* sp. from a salt marsh in the Kuwaiti desert that are capable of degrading crude oil as the sole carbon source at salinity ranging from 5 to 10%. Overall, bacteria, archaea and a few eukaryotes have been shown to degrade crude oil over a broad range of salinity (0–30%). Of these, eubacteria such as *Marinobacter aquaeolei*, *Streptomyces albiaxialis*, and *Actinopolyspora* sp. and archaea such as *Haloferax*, *Halobacterium*, and *Halococcus* withstand extreme salinity (20–30%) and such organisms are important for the cleanup of oil-impacted hypersaline environments since natural attenuation in such environments is too slow (McGenity, 2010).

### Aliphatic compounds

Ward and Brock (1978) carried out some of the earliest experiments on the biodegradation aliphatic compounds including mineral oil and ^14^C-hexadecane in water samples of varying salinity (3.3–28.4% salt) collected at the salt evaporation ponds near the south end of Great Salt Lake (GSL), Utah and also from the middle part of GSL. The authors reported decreasing rates of degradation of mineral oil and ^14^C-hexadecane with increasing salinity up to 20% in natural sample as well in microbial consortium enriched from water samples from GSL. At salinity greater than 20%, degradation was severely inhibited and this lack of degradation was not due to low levels of dissolved oxygen or lack of growth promoting nutrients since both were provided in the experiments. The authors conclude that the rate limitations were probably due to high salinity. Gauthier et al. (1992) have reported the ability of type strain, *Marinobacter hydrocarbonoclasticus* (originally named *Alteromonas* strain sp –17, isolated by Al-Mallah et al. (1990) from hydrocarbon-contaminated sediments in the Mediterranean Sea) to utilize hexadecane (100%), eicosane (91%), and heneicosane (84%), in the presence of 4.6–20% NaCl. In addition, the organism also degraded phenanthrene (41%) and other aliphatics at low levels as single sources of carbon and energy. Later Fernandez-Linares et al. (1996) have studied the effect of various concentrations of NaCl on growth and degradation of eicosane by *M. hydrocarbonoclasticus* and found that an increase in salinity from 1.2 to 14.5% NaCl had no significant effect on eicosane degradation. Huu et al. (1999) have reported the isolation of *Marinobacter aquaeolei* from an oil-producing well in southern Vietnam that degrades *n*-hexadecane and pristane as the sole sources of carbon at 0–20% salinity. Plotnikova et al. (2001) reported degradation of octane as the sole source of carbon in the presence of 6% salt by several gram positive bacteria including *Rhodococcus* sp, *Arthrobacter* sp, and *Bacillus* sp., isolated from sediment samples from chemical- and salt processing plants in Russia. Abed et al. (2006) have shown the biodegradation pristane and *n*-octadecane at salinity ranging from 5 to 12% at temperatures between 15 and 40°C by microbial mats from the coastal flats of the Arabian Gulf. Al-Mueini et al. (2007) have reported the isolation of an extremely halophilic actinomycete, *Actinopolyspora sp.* DPD1 from an oil production site in the Sultanate of Oman and shown to degrade *n*-alkanes (pentadecane, eicosane, pentacoase) and fluorene at 25% salt. The organism efficiently degraded pentadecane (100% in 4 days) and eicosane (80% in 10 days). Degradation of longer chain alkanes such as pentacosane (C_25_H_52_) proceeded at much slower rate resulting in only 15% degradation in 2 weeks and no triacontane (C_30_H_62_) was degraded even after 20 days of incubation. Degradation of fluorine by *Actinopolyspora sp.* DPD1 resulted in several novel intermediates and appears to proceed through previouely undescribed breakdown pathway. The observation that *Actinopolyspora sp.* DPD1 can degrade long chain *n*-alkanes and a polyaromatic hydrocarbon is indicative of its metabolic versatility. Sass et al. (2008) isolated a strain DS-1, closely related to *Bacillus aquimaris* from Discovery deep-sea hypersaline anoxic sediment that grew using *n*-alkanes (*n*-dodecane and *n*-hexadecane) as the sole sources of carbon in the presence of 12–20% NaCl. Mnif et al. (2009, 2011) isolated *Halomonas sp*. strain C2SS100 and *Pseudomonas* sp. strain C450R on the basis of their ability to degrade crude oil also degraded hexadecane as the sole carbon source in the presence of 5–10% NaCl. Dastgheib et al. (2011) have isolated a halotolerant *Alcanivorax* sp. strain Qtet3 from tetracosane degrading enrichments obtained from a hydrocarbon contaminated soils from Qom location in Iran. Strain Qtet3 degrades a wide range of *n*-alkanes (from C_10_ to C_34_) with considerable growth on C_14_ and C_16_ in the presence of 0–15% NaCl. Strain Qtet3 completely degraded tetracosane (C_24_H_50_) as the sole carbon source in 20 days. In addition, the organism also degrades phytane and pristane, but not aromatic hydrocarbons such as naphthalene, phenanthrene, pyrene, and anthracene. As indicated above, two Marinobacters, *M. sedimentalis* and *M. falvimaris* isolated on the basis of their ability to grow on crude oil from hypersaline sabkhas in Kuwait also utilized Tween 80 and a wide range of individual aliphatic hydrocarbons (C_9_–C_40_) as carbon sources in the presence of 6% NaCl (Al-Mailem et al., 2013).

Reports also exist on the ability of archaea that degrade aliphatic hydrocarbons at high salinity. Bertrand et al. (1990) were among the first to report the isolation of a halophilic archaea, strain EH_4_, which was recently classified as *Haloarcula vallismortis* (see Tapilatu et al., 2010) from a salt marsh near the town of Aigues-Mortes in Southern France. The EH_4_ was isolated using agar plates containing eicosane as the sole carbon source. Contrary to the results observed by Ward and Brock (1978) at GSL, the growth of EH_4_ on eicosane increased with increasing salinity. Growth and degradation was maximum at 20% salinity and non-detectable below 10% salinity. Experiments also showed that the isolate was able to degrade a mixture of aliphatic and aromatic hydrocarbons including tetradecane, hexadecane, eicosane, heneicosane, pristane, acenaphtene, phenanthrene, anthracene, and 9-methyl antracene in the presence of >20% NaCl. Kulichevskaya et al. (1992) have reported the isolation of an archaeon, *Halobacterium* that degraded *n*-alkane (C_10_–C_30_) in a medium containing 29% NaCl. Tapilatu et al. (2010) have reported the isolation of several strains of archaea that degrade *n*-alkanes (heptadecane and eicosane) in the presence of 22.5% NaCl from a shallow crystallizer pond (Camargue, France) with no known contamination history. Of these isolates, strain, MSNC 2 was closely related to *Haloarcula* and strains, MSNC 4, MSNC 14, and MSNC 16 to *Haloferax*. In addition, strain MSNC 14 also degraded phenanthrene. Three extremely halophilic archaeal strains, *Haloferax*, *Halobacterium* and *Halococcus* isolated on the basis of crude oil utilization also degraded *n*-alkanes and mono and polyaromatic compounds as the sole sources of carbon and energy in the presence of 26% NaCl (Al-Mailem et al., 2010). Overall, studies reveal that both bacteria and archaea have the capacity to metabolize *n*-alkanes with varying chain lengths in the presence of salt ranging from low to extremely high (Table 2).

**Table 2:** **Degradation of aliphatic hydrocarbons in moderate to high salinity conditions**.

**Hydrocarbon**	**Structure**	**Degrader**	**NaCl (%)**	**References**
Octane	C_8_H_18_	*Rhodococcus* sp. *Arthrobacter* sp. *Bacillus* sp.	6	Plotnikova et al., 2001, 2011
Decane	C_10_H_22_	*Bacillus* sp strain *DHT*	10	Kumar et al., 2007
Tetradecane	C_14_H_30_	EH4 (*Haloarcula vallismortis*)	>20	Bertrand et al., 1990
Pentadecane	C_15_H_32_	*Actinopolyspora* sp.	25	Al-Mueini et al., 2007
		*Marinobacter aquaeolei*	0–20	Huu et al., 1999
Hexadecane	C_16_H_34_	Enrichment, Great Salt Lake, Utah	< 20	Ward and Brock, 1978
		EH4 (*Haloarcula vallismortis*)	>20	Bertrand et al., 1990
		*Marinobacter hydrocarbonoclasticus*	4.6–20	Gauthier et al., 1992
		*Marinobacter aquaeolei*	0–20	Huu et al., 1999
		*Bacillus* sp strain DHT	10	Kumar et al., 2007
		Strain DS1	12–20	Sass et al., 2008
		*Halomonas* sp C2SS100 *Pseudomonas* sp. C450R	10	Mnif et al., 2009
Octadecane	C_18_H_38_	Microbial mats	5–12	Abed et al., 2006
		*Haloferax* sp. *Halobacterium* sp. *Halococcus* sp.	>26	Al-Mailem et al., 2010
Heptadecane	C_17_H_36_	*Haloarcula* sp. *Haloferax* sp.	>22	Tapilatu et al., 2010
Pristane	C_19_H_40_	EH4 (*Haloarcula vallismortis*)	>20	Bertrand et al., 1990
		*Marinobacter hydrocarbonoclasticus*	4.6–20	Gauthier et al., 1992
		*Marinobacter aquaeolei*	0–20	Huu et al., 1999
		Microbial mats, Arabian Gulf coast, Saudi Arabia	5–12	Abed et al., 2006
		*Alcanivorax* sp. Qtet3	0–15	Dastgheib et al., 2011
Eicosane	C_20_H_42_	EH4 (*Haloarcula vallismortis*)	10–20	Bertrand et al., 1990
		*Marinobacter hydrocarbonoclasticus*	1–14	Fernandez-Linares et al., 1996
		*Actinopolyspora* sp. DPD1	25	Al-Mueini et al., 2007
		*Haloarcula* sp. *Haloferax* sp.	>22	Tapilatu et al., 2010
Phytane	C_20_H_42_	*Alcanivorax* sp. strain Qtet3	0–15	Dastgheib et al., 2011
Heneicosane	C_21_H_44_	EH4 (*Haloarcula vallismortis*)	>20	Bertrand et al., 1990
		*Marinobacter hydrocarbonoclasticus*	4.6–20	Gauthier et al., 1992
Tetracosane	C_24_H_50_	*Alcanivorax* sp. strain Qtet3	0–15	Dastgheib et al., 2011
Pentacosane	C_25_H_52_	*Actinopolyspora* sp. DPD1	25	Al-Mueini et al., 2007
n-Alkane	C_10_–C_30_	*Halobacterium* sp	29	Kulichevskaya et al., 1992
	C_10_–C_34_	*Haloferax* sp. *Halobacterium* sp. *Halococcus* sp.	>26	Al-Mailem et al., 2010
	C_10_–C_34_	*Alcanivorax* sp. strain Qtet3	0–15	Dastgheib et al., 2011
	C_9_–C_40_	*Marinobacter sedimentalis Marinobacter falvimaris*	6	Al-Mailem et al., 2013

### Polycyclic aromatic hydrocarbons

Polycyclic aromatic hydrocarbons (PAHs) are ubiquitous in many oily and saline environments. Crude oil contains PAHs containing two to four and five ring-molecules. Because of their toxic, mutagenic, and carcinogenic properties, persistence of PAHs in the environment are of particular concern (Menzie et al., 1992; Gibbs, 1997; Cao et al., 2009). The persistence of PAHs in the environment depends on the number of rings in the molecule and environmental factors such as pH, temperature, and salinity. Although studies have reported the degradation PAHs by non-halophiles and in marine habitats, little is known about the fate of these compounds in high salinity environments. Ashok et al. (1995) have isolated bacterial strains of the genus *Micrococcus, Pseudomonas, and Alcaligenes* from soil samples near an oil refinery that degraded naphthalene and anthracene as the sole sources of carbon at 7.5% salinity. Plotnikova et al. (2001, 2011) have isolated *Pseudomonas* sp., *Rhodococcus* sp., *Arthrobacter* sp., and *Bacillus* sp. from soil and sediment contaminated with waste generated by chemical and salt-producing plants. All these isolates degraded naphthalene and salicylate as the sole carbon sources in the presence of 5–9% NaCl. In addition, some of these organisms also grew on phenanthrene, biphenyl, *o*-phthalate, gentisate, octane, and phenol as the sole sources of carbon. Zhao et al. (2009) have shown the degradation of phenanthrene in the presence of 5–15% NaCl by a halophilic bacterial consortium developed from soil samples collected from the Shengli Oilfield in China. Phenanthrene was completely degraded by the enrichment in 8 days. Molecular analysis of the enrichment culture indicated the presence of *alpha* and *gamma*-*proteobacteria* including members of the genus *Halomonas*, *Chromohalobacter, Alcanivorax, Marinobacter, Idiomarina, and Thalassospira.* Dastgheib et al. (2012) have obtained a mixed culture (Qphe-SubIV) consisting of *Halomonas* sp. and *Marinobacter* sp. from hydrocarbon-contaminated saline soil collected from five different regions in Iran. These organisms degraded several PAHs including naphthalene, phenanthrene, anthracene, fluoranthene, fluorine, pyrene, benz[a]anthracene, and benzo[a]pyrene as the sole carbon sources in the presence of 1–15% NaCl. Recently, Al-Mailem et al. (2013) have reported the ability of *Marinobacter sedimentalis* and *Marinobacter falvimaris* isolated from hypersaline sabkhas to degrade biphenyl, phenanthrene, anthracene and naphthalene as the sole sources of carbon and energy at 6% NaCl. More recently, Gao et al. (2013) have isolated *Marinobacter nanhaiticus* Strain D15-8W from a phenanthrene-degrading enrichment obtained from a sediment from the South China Sea. The strain D15-8W degrades naphthalene, phenanthrene or anthracene as the sole source of carbon in the presence of 0.5–15% with optimum degradation in the presence of 1–5% NaCl.

Studies also show the ability of archaea to degrade PAHs in high salinity environments. As mentioned above, strain EH4 (*Haloarcula vallismortis)*, not only degraded *n*-alkanes but also degraded a mixture of alkanes and aromatic compounds such as acenaphthene, anthracene, and phenathrene at >20% NaCl (Bertrand et al., 1990). Bonfá et al. (2011) have isolated several strains of *Haloferax* that degrade a mixture of the PAHs including naphthalene, anthracene, phenanthrene, pyrene and benzo[a]anthracene at high salinity (20% NaCl). Extremely halophilic archaeal strains of *Haloferax*, *Halobacterium*, and *Halococcus* isolated from a hypersaline coastal area of the Arabian Gulf not only degraded crude oil and *n*-octadecane as the carbon sources, but also grew on phenanthrene at 26% salinity (Al-Mailem et al., 2010). Erdogmuş et al. (2013) showed the degradation of naphthalene, phenanthrene and pyrene as the sole carbon sources in the presence of 20% NaCl by several archaeal strains including *Halobacterium piscisalsi*, *Halorubrum ezzemoulense*, *Halobacterium salinarium*, *Haloarcula hispanica*, *Haloferax* sp. *Halorubrum* sp. and *Haloarcula* sp. isolated from brine samples of Camalt Saltern in Turkey. The hydrocarbon degradation potential of *Halorubrum* sp. and *Halorubrum ezzemoulense* was documented for the first time in this study. These reports clearly demonstrate the potential of bacteria and archaea to degrade PAHs in high salinity environments (Table 3).

**Table 3:** **Biodegradation of polycyclic aromatic hydrocarbons in moderate to high salinity conditions**.

**Hydrocarbon**	**Structure**	**Degrader**	**Salinity (%)**	**References**
Naphthalene	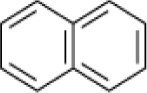	*Micrococcus* sp. *Pseudomonas* sp. *Alcaligenes* sp.	7.5	Ashok et al., 1995
		*Pseudomonas* sp. *Rhodococcus* sp. *Arthrobacter* sp. *Bacillus* sp.	6–9	Plotnikova et al., 2001, 2011
		*Bacillus* sp strain DHT	10	Kumar et al., 2007
		*Haloferax* sp. *Halobacterium* sp. *Halococcus* sp.	>26	Al-Mailem et al., 2010
		*Haloferax* spp.	20	Bonfá et al., 2011
		*Arthrobacter* spp. SN17	6–9	Plotnikova et al., 2011
		Mixed culture (Qphe-SubIV)	1–15	Dastgheib et al., 2012
		*Marinobacter sedimentalis Marinobacter falvimaris*	6	Al-Mailem et al., 2013
		*Marinobacter nanhaiticus*	0.5–15	Gao et al., 2013
		*Halobacterium piscisalsi*, *Halorubrum ezzemoulense*, *Halobacterium salinarium*, *Haloarcula hispanica Haloferax* sp. *Halorubrum* sp. *Haloarcula* sp.	20	Erdogmuş et al., 2013
Anthracene	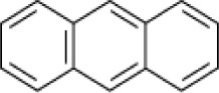	EH4 (*Haloarcula vallismortis)*	>20	Bertrand et al., 1990
		*Micrococcus* sp. *Pseudomonas* sp. *Alcaligenes* sp.	7.5	Ashok et al., 1995
		*Haloferax* spp	20	Bonfá et al., 2011
		Mixed culture (Qphe-SubIV)	1–15	Dastgheib et al., 2012
		*Marinobacter sedimentalis Marinobacter falvimaris*	6	Al-Mailem et al., 2013
		*Marinobacter nanhaiticus*	0.5–15	Gao et al., 2013
Phenanthrene	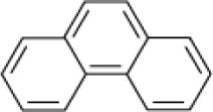	EH4 *(Haloarcula vallismortis)*	>20	Bertrand et al., 1990
		*Micrococcus* sp. *Pseudomonas* sp. *Alcaligenes* sp.	7.5	Ashok et al., 1995
		*Pseudomonas* sp. *Arthrobacter* sp.	5–9	Plotnikova et al., 2001, 2011
		Microbial consortium, Shengli Oilfield, China	5–15	Zhao et al., 2009
		*Haloferax* sp	>22	Tapilatu et al., 2010
		*Haloferax* sp. *Halobacterium* sp. *Halococcus* sp.	>26	Al-Mailem et al., 2010
		*Haloferax* spp.	20	Bonfá et al., 2011
		Mixed culture (Qphe-SubIV)	1–15	Dastgheib et al., 2012
		*Marinobacter sedimentalis Marinobacter falvimaris*	6	Al-Mailem et al., 2013
		*Marinobacter nanhaiticus*	0.5–15	Gao et al., 2013
		*Halobacterium piscisalsi*, *Halorubrum ezzemoulense*, *Halobacterium salinarium*, *Haloarcula hispanica Haloferax* sp. *Halorubrum* sp. *Haloarcula* sp.	20	Erdogmuş et al., 2013
Acenaphthene	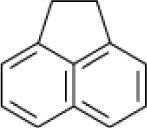	EH4 (*Haloarcula vallismortis)*	>20	Bertrand et al., 1990
Fluorene	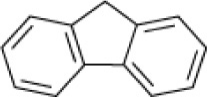	*Actinopolyspora* sp. DPD1	5–20	Al-Mueini et al., 2007
		Mixed culture (Qphe-SubIV)	1–15	Dastgheib et al., 2012
Pyrene	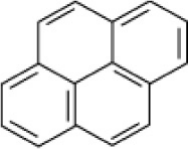	*Bacillus* sp strain DHT	10	Kumar et al., 2007
		*Haloferax* spp.	20	Bonfá et al., 2011
		Mixed culture (Qphe-SubIV)	1–15	Dastgheib et al., 2012
		*Halobacterium piscisalsi*, *Halorubrum ezzemoulense*, *Halobacterium salinarium*, *Haloarcula hispanica Haloferax* sp. *Halorubrum* sp. *Haloarcula* sp.	20	Erdogmuş et al., 2013
Biphenyl	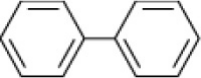	*Rhodococcus* sp. *Arthrobacter* sp.	6–9	Plotnikova et al., 2001, 2011
		*Haloferax* sp. *Halobacterium* sp. *Halococcus*	>26	Al-Mailem et al., 2010
		*Marinobacter sedimentalis Marinobacter falvimaris*	6	Al-Mailem et al., 2013

### Benzene, toluene, ethylbenzene, and xylenes

The most abundant hydrocarbons in produced water are the one-ring aromatic hydrocarbons, benzene, toluene, ethylbenzene, and xylenes (BTEX) and low molecular weight saturated hydrocarbons (Neff et al., 2011). Benzene is a category A carcinogen. Leakage from produced water storage tanks, pipelines, spills, and seepage from surface contaminated sites can cause major BTEX contamination (Philip et al., 2005). BTEX are relatively highly soluble in water and hence can contaminate large volumes of groundwater. Although there have been many recent reports on the biodegradation of non-oxygenated hydrocarbons in moderate to high salinity environments, only few reports exist on the biodegradation of BTEX compounds (Table 4). Nicholson and Fathepure (2004, 2005) have reported the degradation of BTEX at high salinity in microcosms established with soil samples from an oilfield and from an uncontaminated salt flat in Oklahoma. Subsequently, enrichment cultures were obtained from both sites on mineral salts medium containing 14.5% NaCl and benzene as the sole carbon source. The oilfield enrichment degraded BTEX in the presence of 3–14.5% NaCl, whereas the enrichment from the salt flat degraded only benzene and toluene as the sole carbon sources in the presence of 0–23% NaCl. Furthermore, these studies have demonstrated complete mineralization of ^14^C-benzene to ^14^CO_2_ by the enrichment cultures in the presence of 14.5% NaCl. Sei and Fathepure (2009) have developed an enrichment culture using sediment samples from Rozel point in GSL, Utah. The enrichment completely degraded benzene or toluene as the sole source of carbon within 1, 2, and 5 weeks in the presence of 14, 23, and 29% NaCl, respectively. In addition, these authors have successfully isolated two strains of *gamma*-*proteobacteria* identified as *Arhodomonas* sp. strain Seminole (previously referred to as strain SEM-2) and *Arhodomonas* sp. strain Rozel from enrichments developed using a soil sample from an oilfield in Oklahoma and a sediment sample from Rozel Point, respectively (Nicholson and Fathepure, 2006; Azetsu et al., 2009). These strains rapidly degraded benzene and toluene as the sole sources of carbon in the presence of 3–23% NaCl and no degradation was seen at 0 and 30% NaCl. Li et al. (2006) have isolated a *Planococcus* sp. strain ZD22 using a contaminated soil collected from a site near the Daqing oil field in China. The strain ZD22 is a psychrotolerant and moderate haloalkaliphile and degrades BTEX in the presence of 0.5–25% salt. In addition, the strain ZD22 also degraded chlorobenzene, bromobenzene, iodobenzene, and fluorobenzene. This ability of the strain ZD22 to utilize different aromatic compounds, combined with its ability to grow under multiple extreme conditions including low temperature, high salinity, and alkaline pH make it a good candidate for the biodegradation of toxic wastes. Berlendis et al. (2010) have tested the ability of two previousely isolated Marinobacters, *Marinobacter vinifirmus* and *Marinobacter hydrocarbonoclasticus* to degrade BTEX as the sole carbon sources at 3–15% salinity. *M. vinifirmus* was able to degrade all the added benzene and toluene in 3 days, while 65% of total ethylebenzene and 20% of total *p*-xylene were removed in 7 days in the presence of 6% NaCl. Similarly, *M. hydrocarbonoclasticus* degraded 10% of benzene, 20% of toluene, 60% of ethylebezene, and 70% of the added *p*-xylene in 7 days as the sole sources of carbon at 6% salinity. Recently Al-Mailem et al. (2013) have isolated *Marinobacter sedimentalis* and *Marinobacter falvimaris* on the basis of their ability to utilize *n*-alkanes and PAHs. These bacteria were also able to degrade benzene as the sole carbon source in the presence of 6% NaCl thus extending the substrate range for this group of organisms. This is important because Marinobacters are one of the most important groups of halophiles found in a variety of ecosystems ranging from extremely cold to hot, low to high salinity and over a broad range of pH demonstrating their tremendous adaptation capabilities (Duran, 2010). Hassan et al. (2012) have reported the isolation of *Alcanivorax* sp. HA03 from soda lakes in Wadi E1Natrun capable of degrading benzene, toluene, and chlorobenzene as the sole sources of carbon at salinity ranging from 3 to 15% NaCl. This observation that Alcanivorax can also degrade aromatic compounds expands the metabolic capability of this group of organisms because Alcanivorax are primarily known for their ability to degrade aliphatic hydrocarbons. Degradation of benzene was also reported in archaea. For example, the crude oil degrading *Haloferax*, *Halobacterium*, and *Halococcus* isolated from a hypersaline Arabian Gulf coast degraded benzene as the sole source of carbon at 26% salinity (Al-Mailem et al., 2010). As mentioned above, to date, only few microoganisms have been shown to degrade BTEX in moderate to high salinity conditions. This is not surprising considering that BTEX are volatile compounds and lack an activating oxygen or nitrate moiety thus making these compounds less available and resistant to biodegradation.

**Table 4:** **Degradation of benzene, toluene, ethylbenzene and xylenes in moderate to high salinity conditions**.

**Hydrocarbon**	**Structure**	**Degrader**	**Salinity (%)**	**References**
Benzene	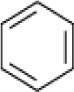	Enrichment, Oilfield Oklahoma	3–14	Nicholson and Fathepure, 2004
		Enrichment, Great Salt Plains, Oklahoma	0–23	Nicholson and Fathepure, 2005
		*Planococcus* sp. strain ZD22	5–20	Li et al., 2006
		*Arhodomonas* sp. strain Seminole	3–17	Nicholson and Fathepure, 2006; Dalvi et al., 2012
		Enrichment, Rozel Point, Great Salt Lake, Utah	0–29	Sei and Fathepure, 2009
		*Arhodomonas* sp. strain Rozel	3–23	Azetsu et al., 2009; Dalvi et al., 2012
		*Marinobacter vinifirmus, M. hydrocarbonoclasticus*	3–15	Berlendis et al., 2010
		*Haloferax* sp. *Halobacterium* sp. *Halococcus* sp.	>26	Al-Mailem et al., 2010
		*Alcanivorax* sp.HA03	3–15	Hassan et al., 2012
		*Marinobacter sedimentalis*, *Marinobacter falvimaris*	6	Al-Mailem et al., 2013
Toluene	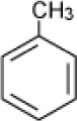	Enrichment, oilfield soil, Oklahoma	3–14	Nicholson and Fathepure, 2004
		Enrichment, Great Salt Plains, Oklahoma	0–23	Nicholson and Fathepure, 2005
		*Planococcus* sp. strain ZD22	5–20	Li et al., 2006
		*Arhodomonas* sp. strain Seminole	3–17	Nicholson and Fathepure, 2006; Dalvi et al., 2012
		Enrichment, Rozel Point, Great Salt Plains, Utah	0–29	Sei and Fathepure, 2009
		*Arhodomonas* sp. strain Rozel	3–23	Azetsu et al., 2009; Dalvi et al., 2012
		*Marinobacter vinifirmus, M. hydrocarbonoclasticus*	3–15	Berlendis et al., 2010
		*Haloferax* sp. *Halobacterium* sp. *Halococcus sp.*	>26	Al-Mailem et al., 2010
		*Alcanivorax* sp.HA03	3–15	Hassan et al., 2012
Ethylbenzene	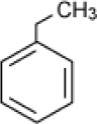	Enrichment, oilfield soil, Oklahoma	3–14	Nicholson and Fathepure, 2004
		*Planococcus* sp. strain ZD22	5–20	Li et al., 2006
		*Marinobacter vinifirmus, M. hydrocarbonoclasticus*	3–15	Berlendis et al., 2010
Xylene	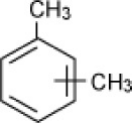	Enrichment, oilfield soil, Oklahoma	3–15	Nicholson and Fathepure, 2004
		*Planococcus* sp. strain ZD22	5–20	Li et al., 2006
		*Marinobacter vinifirmus, M. hydrocarbonoclasticus*	3–15	Berlendis et al., 2010

### Phenolics and benzoates

Industrial effluents generated from many food, dye, pharmaceutical, and chemical processing operations are often characterized by high salinity and the presence of phenolics and benzoates (Garcia et al., 2005b). In addition, compounds such as 4-hydroxybenzoic, ferulic, *p*-coumaric, vanillic, cinnamic, and syringic acids are naturally present in lignin and plant root exudates (Le Borgne et al., 2008). In recent years, many studies have successfully isolated bacteria and archaea that degrade oxygenated aromatics in saline conditions. Table 5 lists organisms that degrade oxygenated hydrocarbons in moderate to high salinity conditions.

**Table 5:** **Biodegradation of phenolics and benzoates in moderate to high salinity conditions**.

**Hydrocarbon**	**Structure**	**Degrader**	**Salinity (%)**	**References**
Phenol	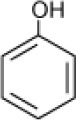	Halophilic isolate	1–15	Woolard and Irvine, 1995
		*Halomonas* sp.	1–14	Hinteregger and Streischsberg, 1997
		*Candida tropicals*	15	Bastos et al., 2000
		*Halomonas campisalis*	0–15	Alva and Peyton, 2003
		*Halomonas organivorans*	1.5–30	Garcia et al., 2004, 2005b
		*Thelassobacillus devorans*	7.5–10	Garcia et al., 2005a
		*Arthrobacter* sp.	6–9	Plotnikova et al., 2011
		*Halomonas organivorans*, *Arhodomonas aquaeolei*, *Modicisalibacter tunisiensis*	10	Bonfá et al., 2013
		Strain C5	12	Chamkha et al., 2008
Benzoate	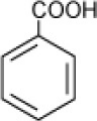	*Halomonas halodurans*	15	Rosenberg, 1983
		*Haloferax* sp. D1227	15	Emerson et al., 1994
		*Halomonas* sp.	35	Kleinsteuber et al., 2001
		*Halomonas organivorans*	1.5–30	Garcia et al., 2004, 2005b
		*Halomonas elongate Halomonas eurihalina Marinobacter lipolyticus*	10	Garcia et al., 2005b
		*Halomonas campisalis*	5–10	Oie et al., 2007
		*Chromohalobacter* sp. strain HS-2	10	Kim et al., 2008
		*Haloferax* sp.	20	Bonfá et al., 2011
		Strain C5	12	Chamkha et al., 2008
Salicylate	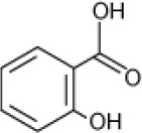	*Pseudomonas* sp. *Rhodococcus* sp. *Arthrobacter* sp. *Bacillus* sp.	5–9	Plotnikova et al., 2001
		*Halomonas organivorans*	1.5–30	Garcia et al., 2004
		*Halomonas organivorans, Salinicoccus roseus Halomonas venusta Halomonas alimentaria*	10	Garcia et al., 2005b
		*Halomonas campisalis*	5–10	Oie et al., 2007
		*Bacillus* sp. strain DHT	10	Kumar et al., 2007
		*Haloferax* sp.	20	Bonfá et al., 2011
*o*-Phthalate	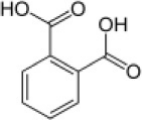	*Rhodococcus* sp. *Arthrobacter* sp. *Bacillus* sp.	5–9	Plotnikova et al., 2001, 2011
Gentisate	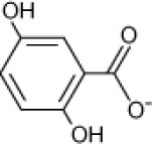	*Rhodococcus* sp. *Arthrobacter* sp. *Bacillus* sp.	3–29	Plotnikova et al., 2001
4-Hydroxy-benzoate	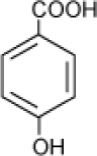	*Haloarcula* sp. strain D1,	17	Fairley et al., 2002
		*Halomonas organivorans*	1.5–30	Garcia et al., 2004
		*Halomonas elongate*	10	Garcia et al., 2005b
		Halophilic archaeal strains	20	Cuadros-Orellana et al., 2006, 2012
		*Chromohalobacter* sp. strain HS-2	10	Kim et al., 2008
		Strain C5	12	Chamkha et al., 2008
		*Haloferax* sp.	20	Bonfá et al., 2011
		*Halobacterium* sp. *Haloferax* sp. *Halorubrum* sp. *Haloarcula* sp.	20	Erdogmuş et al., 2013
Phenyl propionic acid	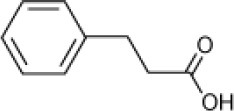	*Haloferax* sp. D1227	5–29	Emerson et al., 1994; Fu and Oriel, 1999
		*Halomonas organivorans Halomonas elongata Halomonas glaciei*	10	Garcia et al., 2005b
		*Halomonas organivorans*	1.5–30	Garcia et al., 2004, 2005b
Cinnamic acid	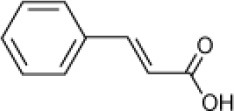	*Haloferax* sp. D1227	5–30	Emerson et al., 1994
		*Halomonas organivorans*	1.5–30	Garcia et al., 2004
		*Halomonas organivorans Halomonas salina/Halomonas halophila Halomonas elongate*	10	Garcia et al., 2005b
		*Halomonas* strain IMPC	0–25	Abdelkafi et al., 2006
		Strain C5	12	Chamkha et al., 2008
*p*-Coumaric acid	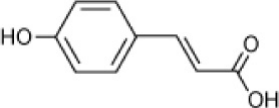	*Halomonas organivorans*	1.5–30	Garcia et al., 2004
		*Halomonas organivorans Halomonas salina Chromohalobacter israelensis*	10	Garcia et al., 2005b
		*Halomonas* strain IMPC	0–25	Abdelkafi et al., 2006
		Strain C5	12	Chamkha et al., 2008
Ferulic acid	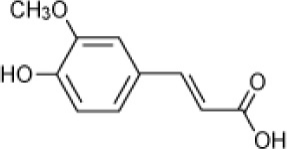	*Halomonas organivorans*	1.5–30	Garcia et al., 2004
		*Halomonas* strain IMPC	0–25	Abdelkafi et al., 2006
		*Halomonas elongate*	10	Garcia et al., 2005b
		Strain C5	12	Chamkha et al., 2008

Woolard and Irvine (1995) showed that a halophile isolated from a mixed culture obtained from a saltern at GSL, Utah readily degraded phenol in the presence of 1–15% NaCl. Similarly, Hinteregger and Streischsberg (1997) reported that a *Halomonas* sp. isolated from a co-culture developed from GSL degraded phenol as the sole source of carbon in the presence of 1–14% salt. Complete degradation of phenol occurred in 13 h at 5% NaCl but at higher NaCl concentrations, degradation occurred with longer lag periods. For example, at 14% NaCl, phenol was completely removed with a lag of 100 h. The degradation of phenol in this organism was accompanied by the accumulation of *cis*, *cis*-muconic acid, a product of *ortho*-cleavage pathway by catechol 1, 2-dioxygenase enzyme. Bastos et al. (2000) reported the isolation of a yeast, *Candida tropicalis* from an enrichment developed from Amazonian rain forest soil that degraded phenol in the presence of up to 15% NaCl. Alva and Peyton (2003) isolated a haloalkaliphile, *Halomonas campisalis* near Soap Lake in central Washington and showed that this organism degraded phenol and catechol as the sole sources of carbon at pH 8–11 and salinity of 0–15%. Formation of metabolic intermediates such as catechol and *cis*, *cis*-muconic acid suggests that phenol was degraded by the *ortho*-cleavage pathway of the beta-ketoadipate branch. A Gram-positive halophilic bacterium, *Thalassobacillus devorans* isolated from an enrichment culture developed from saline habitats in southern Spain was shown to degrade phenol (Garcia et al., 2005a) in the presence of 7.5–10% NaCl. The strain C5, closely related to *Geobacillus pallidus* isolated from a tyrosol-utilizing enrichment also degrades a variety of other oxygenated aromatic compounds including benzoic, *p-hydroxybenzoic*, protocatechuic, vanillic, *p-hydroxyphenylacetic*, 3,4-dihydroxyphenylacetic, cinnamic, ferulic, phenol, and *m*-cresol. However, no degradation of non-oxygenated hydrocarbons such as toluene, naphthalene, and phenanthrene was observed (Chamkha et al., 2008). Recently, Bonfá et al. (2013) have shown the degradation of phenol as the sole source of carbon in the presence of 10% NaCl by *Halomonas organivorans*, *Arhodomonas aquaeolei*, and *Modicisalibacter tunisiensis* isolated from different hypersaline environments.

Many reports also exist on the ability of halophilic and halotolerant organisms to degrade benzoates in high salinity conditions. The halotolerant, *Pseudomonas halodurans* (reclassified as *Halomonas halodurans*) degrades benzoic acid in the presence of >15% NaCl (Rosenberg, 1983). Garcia et al. (2004, 2005b) have isolated several strains of *Halomonas* spp. including the *Halomonas organivorans* from water and sediment of salterns and hypersaline soils collected in different part of the Southern Spain with salinity of the sampling site ranging from 4 to 17%. These isolates degraded a wide range of aromatic compounds including benzoic acid, *p*-hydroxybenzoic acid, phenol, salicylic acid, *p*-aminosalicylic acid, phenylacetic acid, phenylpropionic acid, cinnamic acid, ferulic acid, and *p*-coumaric acid as the sole sources of carbon in the presence of 10% NaCl. Abdelkafi et al. (2006) have reported the isolation of a *p*-coumaric acid degrading *Halomonas* strain IMPC from a *p*-coumaric acid degrading enrichment culture obtained from a table-olive fermentation rich in aromatic compounds. This strain converted *p*-coumaric acid to *p*-hydroxybenzaldehyde, *p*-hydroxybenzoic acid, and then to protocatechuic acid prior to ring cleavage in the presence of 0–25% NaCl. In addition, the strain also degraded other lignin-related compounds such as cinnamic acid, *m*-coumaric acid, *m- and p*-methoxycinnamic acid, *m*- and *p*-methylcinnamic acid, and ferulic acid to their corresponding benzoic acid derivatives. Oie et al. (2007) have studied the degradation of benzoate and salicylate by *Halomonas campisalis* isolated from an alkaline Soap Lake in the presence of 5–10% NaCl. This study showed that the organism degraded benzoate and salicylate to catechol and then to *cis*, *cis*-muconate thus indicating degradation *via* the *ortho*-cleavage pathway. Kim et al. (2008) have isolated a *Chromohalobacter* sp. strain HS-2 from salted fermented clams that degrades benzoate and *p*-hydroxybenzoate at 10% NaCl as the sole carbon and energy sources.

Studies have also documented aerobic degradation of benzoates by extremely halophilic archaea, often growing in near-saturated brines (>30% NaCl). For example, Emerson et al. (1994) isolated a *Haloferax* sp. D1227 from an oil-brine soil near Grand Rapids, Michigan and was shown to degrade benzoic acid, 3-hydroxybenzoic acid, 3-phenylpropionic acid, and cinnamic acid as the sole sources of carbon at salt concentration ranging from 5 to 30% NaCl. When grown on ^14^C-benzoate, strain D1227 conversted 70% of the substrate to ^14^CO_2_ and assimilated 19% of the ^14^C-label into cell biomass. These compounds were degraded *via* a gentisate pathway (Fu and Oriel, 1998, 1999). Fairley et al. (2002) have isolated a novel halophilic archaeon, *Haloarcula* sp. D1 from a high salt enrichment culture and shown to degrade *p*-hydroxybenzoic acid as the sole source of carbon. Cuadros-Orellana et al. (2006) have isolated 44 archaeal strains from five geographically different saline environments including the Uyuni salt marsh in Bolivia, solar saltern in Chile, solar saltern in Puerto Rico, Dead Sea near Jordan, and sabkhas in Saudi Arabia. Analysis of lipid composition and restriction analysis of 16S rDNA-gene places all the strain in four groups in the Halobacteriaceae family. These strains degraded *p*-hydroxybenzoic acid as the sole carbon source in the presence of 20% NaCl. Similarly, Bonfá et al. (2011) have isolated 10 halophilic archaea, all belonging to the genus *Haloferax* from *p*-hydroxybenzoic acid -utilizing mixed cultures obtained from the above five hypersaline sites. These strains were also able to degrade a mixture of *p*-hydroxybenzoic acid, benzoic acid, and salicylic acid as growth substrates in a medium containing 20% NaCl. Recently, Cuadros-Orellana et al. (2012) have reported the isolation of 10 halophilic archaea from Dead Sea that degrade *p*-hydroxybenzoic acid as the sole carbon and energy source. In addition, strain L1, a member of the unclassified Halobacteriaceae family of the phylum, *Euryarchaeota* also degrades benzoic acid to gentisate. Erdogmuş et al. (2013) reported the ability of many archaeal strains belonging to *Halobacterium*, *Haloferax*, *Halorubrum*, and *Haloarcula* group to degrade *p*-hydroxybenzoic acid in a medium containing 20% NaCl. These studies clearly demonstrate that archaea that metabolize *p*-hydroxybenzoic are widespread in the environment. Among bacteria, *Halomonas* spp. have been frequently reported for their ability to degrade phenolics and benzoates and only few reports exist on their potential to degrade non-oxygenated hydrocarbons. Therefore, to fully realize their remediation potential, more studies are needed to determine their capacity to degrade BTEX and PAHs.

## Molecular mechanism of hydrocarbon degradation in high salinity environment

In the last two decades there has been impressive progress in the area of hydrocarbon degradation in hypersaline environments. Pure cultures of aerobic bacteria, archaea, and some eukaryotes have been isolated that degrade hydrocarbons over a broad range of salinities. However, similar progress on genetics and biochemistry of hydrocarbon degradation is severely lacking. Extensive information exists in the literature on the degradation pathways and enzymes involved in the aerobic metabolism of petroleum compounds for many non-halophiles (Reineke, 2001; Van Hamme et al., 2003; Cao et al., 2009). In non-halophiles, monooxygenases initiate degradation of aliphatic hydrocarbons by the addition of oxygen atom (s) to the terminal or subterminal carbon and converting them to corresponding fatty acids which are then assimilated *via* beta-oxidation (Patzelt, 2007). The integral-membrane non-heme di-iron monooxygenase (*Alk*B) and the cytochrome P450 CYP153 family alkane hydroxylases (van Beilen and Funhoff, 2007) catalyze the hydroxylation of medium-chain-length alkanes (C_8_–C_16_), while a flavin-binding monooxygenase (*Alm*A) and a long chain alkane monooxygenase (*Lad*A) have shown to be responsible for the degradation of long chain alkanes with chain length >C_18_ (Feng et al., 2007; Throne-Holst et al., 2007).

Similarly, a wide variety of aromatic hydrocarbons are degraded by monooxygenases or dioxygenases by the addition of oxygen atom (s) to the alkyl moiety or aromatic ring (Reineke, 2001; Van Hamme et al., 2003; Cao et al., 2009; Pérez-Pantoja et al., 2010) converting them to a few central intermediates such as catechols, protocatechuate, and gentisate through convergent pathways. These ring intermediates are cleaved by *ortho*- or *meta*-cleavage dioxygenases such as catechol 1, 2-dioxygenase (1,2-CAT), catechol 2,3-dioxygenase (2,3-CAT), protocatechuate 3,4-dioxygenases (3,4-PCA), and protocatechuate 4,5-dioxygenase (4,5-PCA), and gentisate 1,2-dioxygenase (1,2-GDO) enzymes (Lack, 1959; Harwood and Parales, 1996; Reineke, 2001) into intermediary metabolites such as acetyl Co-A, succinyl Co-A, and pyruvate that feed into the Kreb cycle (Fuchs et al., 2011). The genes encoding these enzymes have been characterized for a variety of aerobic microorganisms including several members the genera *Pesudomonas*, *Rhodococcus*, *Ralstonia*, and *Mycobacterium*, *Acinetobacter* (Luz et al., 2004; Cao et al., 2009).

To date little information exists about the pathways and enzymes for hydrocarbon degradation in high salinity environments. A few recent studies have shown that the degradation of hydrocarbons at high salinity occurs using enzymes described for many non-halophiles. For example, detection of ring-oxidation and ring-cleavage intermediates such as catechol and *cis*-, *cis*-muconate in benzoate and phenol degrading *Halomonas* spp. indicate the role of *ortho*-cleaving enzymes in the beta-ketoadipate pathway for aromatic metabolism (Hinteregger and Streischsberg, 1997; Alva and Peyton, 2003; Oie et al., 2007). Garcia et al. (2005b) have used PCR and degenerate primers for the detection of genes that code for 1,2-CAT and 3,4-PCA enzymes in several strains of phenol- and benzoate degrading *Halomonas* spp. Furthermore, activity of these enzymes was measured in cell free extract of *Halomonas organivorans* cells grown on various aromatic compounds. Recently, Moreno et al. (2011) have further characterized the genes involved in the metabolism of phenol and benzoate in *Halomonas organivorans* in much detail. The gene cluster *catRBCA* involved in the utilization of catechol was isolated from *H*. *organivorans*. The genes *cat*A, *cat*B, *cat*C, and *cat*R that encode for 1,2-CAT, *cis*,*cis*-muconate cycloisomerase, muconolactone delta-isomerase and a LysR-type transcriptional regulator, respectively, were detected. Downstream of these genes were flanked by the benzoate catabolic genes, *ben*A and *ben*B that code for large and small subunit of benzoate 1, 2 dioxygenase, respectively. This gene organization in *H*. *organivorans* was found to be similar to that of the catabolic genes identified in other non-halophilic eubacteria. Abdelkafi et al. (2006) studied the metabolism of *p*-coumaric acid by *Halomonas* strain IMPC under halophilic conditions. Strain IMPC degraded *p*-coumaric acid to *p*-hydroxybenzaldehyde, *p*-hydroxybenzoic acid and then to protocatechuic acid as the final aromatic product before ring fission. The identity of these intermediates was confirmed using a gas chromatography and mass-spectrometry (GC-MS). Kim et al. (2008) isolated a benzoate- and *p*-hydroxybenzoate metabolizing halophile, *Chromohalobacter* sp. strain HS-2. Using a combination of molecular and biochemical approaches, these researchers have elucidated the catabolic pathways for benzoate and *p*-hydroxybenzoate in HS-2. Their work showed that benzoate induces the expression of benzoate 1,2-dioxygenase, 1,2-CAT, *p*-hydroxybenzoate hydroxylase (*pob*A), and 3,4-PCA, while *p*-hydroxybenzoate only induced the expression of *pob*A. Interestingly, the role of *pob*A and 3,4-PCA genes in benzoate grown HS-2 cells is not clear because benzoate is usually degraded *via* catechol by 1,2-CAT or 2,3-CAT. Dastgheib et al. (2011) have obtained a mixed culture, Qphe-SubIV consisting of only two organisms, *Halomonas* sp and *Marinobacter* sp. These organisms degrade phenanthrene. Metabolite analysis showed that 2-hydroxy 1-naphthoic acid and 2-naphthol were among the major metabolites accumulated in the culture media, indicating that an initial dioxygenation step might have proceed by a novel mechanism at C1 and C2 positions. Recently, Dalvi et al. (2012) have analyzed the draft genome sequence of the extremely halophilic benzene and toluene degrading *Arhodomonas sp.* strain Seminole. The analysis predicted 13 putative genes that encode upper and lower pathway enzymes for aromatic compound degradation. These proteins share 44–77% sequence identity with proteins previously described in non-halophilic organisms. The results indicate that benzene is converted to phenol and then to catechol in two steps by monooxygenase-like enzymes closely related to phenol hydroxylases. Thus, formed catechol undergoes ring cleavage *via* the *meta* pathway by 2, 3-CAT to form 2-hydroxymuconic semialdehyde, which subsequently enters the tricarboxylic acid cycle. To corroborate these predicted enzymes that benzene is converted to first phenol and then to catechol prior to ring cleavage by 2,3-CAT, the authors grew a closely related species *Arhodomonas sp.* strain Rozel on deuterated benzene and deuterated phenol was detected by GC-MS as the initial intermediate of benzene degradation. A 2-D gel electrophoresis and Tandem mass-spectrometry has identified the phenol hydroxylase-like and 2,3-CAT in the cell extract of strain Rozel grown on benzene as the sole carbon source. More recently, Bonfá et al. (2013) showed the presence of 1,2-CTD and 3,4-PCD genes in three of phenol degrading bacteria, *Halomonas organivorans*, *Arhodomonas aquaeolei*, and *Modicisalibacter tunisie.*

A few recent studies have provided information about aliphatic hydrocarbon degradation in saline environments. Dastgheib et al. (2011) have used PCR and degenerate primers to amplify two putative *alk* B genes that code for alkane hydroxylases needed for the hydroxylation of aliphatic hydrocarbons in *Alcanivorax* sp. strain Qtet3. The strain Qtet3 degrades a wide range of *n-alkanes* in the presence of 0–15% NaCl. More recently, Nie et al. (2013) have analyzed the full genome of the alkane-metabolizing *Amycolicicoccus subflavus* isolated from an oily sludge at Daqing Oilfield, China (Wang et al., 2010). The organism grew utilizing C_10_–C_36_ alkanes as the sole sources of carbon in the presence of 1–12% NaCl. Four types of alkane hydroxylase coding genes were identified in the genome. A quantitative real-time reverse transcription PCR was used to determine the induction of various alkane-degrading genes. Homologs of *Alk*B alkane hydroxylases were induced by C_10_–C_36_ alkanes with maximum expression in the presence of C_16_–C_24_. Similarly, cytochrome P450 CYP153 genes were upregulated by alkanes, C_10_–C_20_ and C_24_. In addition, *Lad*A and propane monooxygenase genes responsible for the oxidation of C_16_–C_36_ and propane, respectively, were also detected. Interestingly, analysis showed that key genes necessary for the degradation of aromatic compounds were missing in the genome. These physiological, genomic, and transcriptional analyses clearly reveals the *Amycolicicocus subflavus*'s potential to utilize a range of *n*-alkanes typically found in crude oil.

A few reports also exist in the literature on the degradation mechanism of hydrocarbons by archaea in the presence of high salt. For example, an extremely halophilic archaeon, *Haloferax* sp. strain D1227 that degrades benzoate, cinnamate, and phenylpropanoate, was shown to possess 1,2-GDO (Emerson et al., 1994; Fu and Oriel, 1998). Fairley et al. (2002, 2006) also found a closely related gene encoding 1,2-GDO in 4-hydroxybenzoate-degrading *Haloarcula* sp. strain D1. A recent study reported the isolation of nine archaeal isolates belonging to various genera that degraded *p-hydroxybenzoate*, naphthalene, phenanthrene, and pyrene as the sole carbon and energy sources in the presence of 20% NaCl. This study showed that the isolates possessed genes that encode 1,2-CAT and 3,4-PCA and the expression of these genes was measured spectrophotometrically (Erdogmuş et al., 2013).

Overall, these recent few studies show that microorganisms in high-salinity environments degrade hydrocarbons using enzymes and steps similar to those found in non-halophiles. However, in-depth studies are needed to obtain greater insights into degradation pathways and steps leading to intermediates that enter central metabolism. In addition, molecular studies can help develop specific probes to identify and monitor specific degradative organisms in the environment and their *in-situ* activity.

## Conclusions

As summarized in this review, knowledge on the ability of microorganisms capable of degrading hydrocarbons in hypersaline environments has accumulated significantly in the past two decades. Studies show that much richer microbial diversity exists in the environment that can efficiently degrade hydrocarbons over a broad range of salinity. Among microbial taxa, members of the genus *Halomonas*, *Marinobacter*, and *Alcanivorax* are common inhabitants of high salinity environments with the potential to degrade variety hydrocarbons. Among archaea, *Heloferax*, *Haloarchula*, and *Halobacterium* seem to play important role in the degradation of hydrocarbons, especially in extremely high salinity conditions. The implication that pigment-mediated ATP synthesis help archaea better survive and degrade hydrocarbons in oxygen deficient hypersaline environments explains the dominance of these organisms in such environments. Martins and Peixoto (2012) have suggested that halophilic photoautotrophs can be a critical factor for the degradation of hydrocarbons since their activity could compensate the lack of oxygen imposed by hypersalinity. In any case, the synergistic interactions between photosynthetic organisms and hydrocarbon degrading bacteria or archaea can lead to effective biodegradation of hydrocarbons in hypersaline environments is noteworthy and needs further investigation. Studies have revealed that many organisms are capable of degrading a mixture of hydrocarbons in widely fluctuating salinities and some produce surfactants and also some fix nitrogen thus underscoring the importance of such microbes in the cleanup of contaminated sites. Though appreciable progress has been made recently in understanding diversity of microorganisms responsible for hydrocarbon degradation under aerobic conditions, similar information under anaerobic condition is lacking. Also, information on genes, enzymes and molecular mechanism of hydrocarbon degradation, in high salinity environments is not fully understood. A few recent studies have shown that the degradation of hydrocarbons in moderate and to high salinity occurs using enzymes described for many non-halophiles. Recent advances in high-throughput DNA sequencing are providing new tools and capabilities for discovering novel hydrocarbon-degrading microorganisms, especially with new dioxygenases. A better knowledge of the diversity of catabolic pathways would certainly bring valuable information for the development of robust bioremediation processes for hypersaline environments.

### Conflict of interest statement

The author declares that the research was conducted in the absence of any commercial or financial relationships that could be construed as a potential conflict of interest.
